# Emergency Department Visits for Heat Stroke in the United States, 2009 and 2010

**DOI:** 10.1186/2197-1714-1-8

**Published:** 2014-04-24

**Authors:** Xian Wu, Joanne E Brady, Henry Rosenberg, Guohua Li

**Affiliations:** 1 Department of Epidemiology, Columbia University Mailman School of Public Health, New York, NY USA; 2 Department of Anesthesiology, Columbia University College of Physicians and Surgeons, New York, NY USA; 3 Department of Medical Education and Clinical Research, Saint Barnabas Medical Center, Livingston, NJ USA; 4 Malignant Hyperthermia Association of the United States, Sherburne, NY USA; 5 Center for Injury Epidemiology and Prevention, Columbia University Medical Center, 722 W. 168th Street, Room 524, New York, 10032 NY USA

**Keywords:** Emergency medical service, Epidemiology, Global warming, Heat stroke, Public health

## Abstract

**Background:**

The effect of extreme heat on health has become a growing public health concern due to climate change. We aimed to examine the epidemiological patterns of hospital-based emergency department (ED) visits for heat stroke in the United States.

**Findings:**

We analyzed data from the 2009 and 2010 Nationwide Emergency Department Sample, the largest ED data system sponsored by the Agency for Healthcare Research and Quality. ED visits for heat stroke were identified by screening the recorded diagnoses using the International Classification of Diseases, Ninth Revision, Clinical Modification (ICD-9-CM) code 992.0. Annual incidence rates of ED visits for heat stroke were computed according to demographic characteristics and geographic regions. In 2009 and 2010, there were an estimated 8,251 ED visits for heat stroke in the United States, yielding an annual incidence rate of 1.34 visits per 100,000 population (95% Confidence Interval [CI] = 1.23-1.45). Significantly higher incidence rates were found in males (1.99 per 100,000; 95% CI = 1.82-2.16), adults aged ≥ 80 years (4.45 per 100,000; 95% CI = 3.73-5.18), and residents living in the southern region (1.61 per 100,000; 95% CI = 1.43-1.79). The majority (63.1%) of ED visits for heat stroke occurred during the summer months of June, July and August. Over one-half (54.6%) of the ED visits for heat stroke required hospitalization and 3.5% of the patients died in the ED or hospital.

**Conclusions:**

Heat stroke results in approximately 4,100 ED visits each year in the United States, with the majority occurring in the summer months and requiring admission to the hospital. Men, the elderly, and people living in the south region are at heightened risk.

**Electronic supplementary material:**

The online version of this article (doi:10.1186/2197-1714-1-8) contains supplementary material, which is available to authorized users.

## Introduction

Heat stroke is a severe form of heat illness with potentially grave outcomes (Knowlton et al. 2009). Clinically, heat stroke is defined as a core body temperature that rises above 40°C, accompanied by hot, dry skin and central nervous system abnormalities such as delirium, convulsions, or coma (Bouchama and Knochel 2002). Heat stroke is characterized by a patient’s inability to transfer heat produced from normal metabolic activity or exercise to the environment, which results in hyperthermia and cellular injury (Jardine 2007). Hyperthermia can be extremely harmful and potentially fatal because a prolonged elevation in core temperature -- over 40°C lasting 30–60 minutes -- may exceed the ability for the cells to tolerate the thermal stress leading to multi-organ tissue damage (Kerr et al. 2013). Therefore, heat stroke often requires emergency care in order to obtain prompt and aggressive interventions and minimize long-term consequences.

Exposure to extreme heat is the main cause of heat stroke, especially during heat waves (Changnon et al. 1996). Predisposing factors of heat stroke include high ambient temperature, intense solar radiation, low level of physical fitness, sleep deprivation (Casa et al. 2012a), social isolation (Semenza et al. 1996), and lower access to air conditioning (O'Neill et al. 2005). Elderly adults and people with cardiovascular and respiratory disease, diabetes, obesity and the use of medications that reduce sweating, impair thermoregulation, and weaken cardiovascular response are at higher risk from heat stroke (Bonauto et al. 2007). With increasing frequency, duration, and intensity of heat waves both globally and in the United States, heat stroke could become a health threat to a larger population in the future and pose a greater burden on the healthcare system (Meehl and Tebaldi 2004).

Previous epidemiological studies of heat stroke or other heat-related illness were limited to selected population groups, such as athletes, mining workers and military personnel, and to outbreaks during heat waves (Bonauto et al. 2007; Semenza et al. 1996; Casa et al. 2012b; Armed Forces Health Surveillance Center 2013; Hunt et al. 2013). Little is known about the incidence and epidemiological patterns of heat stroke in the general population at a national level. We sought to close this research gap by drawing on recently available data from hospital-based emergency departments (EDs) in the United States. Information about the epidemiologic patterns of ED visits for heat stroke may help assist public health professionals and emergency medical personnel to identify high-risk population groups and develop effective preventive strategies and clinical interventions.

## Findings

### Methods

#### Data source

Data for this study came from the Nationwide Emergency Department Sample (NEDS) for 2009 and 2010. The NEDS dataset is part of the Healthcare Cost and Utilization Project sponsored by the Agency for Healthcare Research and Quality. NEDS is the largest all-payer ED database, containing approximately a 20% stratified sample of all US hospital-based EDs and (Agency for Healthcare Research and Quality 2014a). In 2009, 964 hospitals in 29 states contributed data to NEDS and in 2010, 961 hospitals in 28 states contributed data to NEDS. This database captures discharge information for ED visits as well as inpatient information on ED patients admitted into the same hospital. NEDS records data for each visit on more than 100 variables, including patient demographic characteristics, hospital characteristics, up to 19 diagnoses documented during the ED visit and subsequent hospitalization, procedures performed during hospitalization, disposition from the ED, insurance status and total charges for ED service.

NEDS uses a stratified, single-stage cluster sampling procedure to obtain a nationally representative sample of hospital-based ED visits. Sample stratification is based on five hospital characteristics: geographic region, trauma center designation, urban–rural location of the hospital, teaching status and hospital ownership type. Sampling weights assigned to individual visits allow for national estimates of all hospital-based ED visits. Population data from the US Census Bureau for July 1, 2009, and April 1, 2010 were added up as the denominator when calculating incidence rates, which were expressed as ED visits for heat stroke per 100,000 population per year (U.S. Census Bureau 2010, 2011).

#### Study variables

For each visit, up to 19 diagnoses were recorded and coded with the International Classification of Diseases, Ninth Revision, Clinical Modification (ICD-9-CM) codes. ED visits for heat stroke and sun stroke were identified by screening for the corresponding ICD-9-CM code (992.0) in any of the nineteen recorded diagnoses. Excluded from the study were other heat-related illnesses (ICD-9-CM codes 992.1-992.9), such as heat syncope, heat cramps, and heat exhaustion, which are less severe than heat stroke and are likely more susceptible than heat stroke to biases from socioeconomic status, healthcare seeking behavior, and misclassification. Age in years was categorized into the following groupings: 0–19, 20–29, 30–39, 40–49, 50–59, 60–69, 70–79 and ≥ 80. We also assessed the following variables: sex, urban–rural status, month of visit, diagnosis, and disposition from the ED. Urban–rural status was based on the Office of Management and Budget (OMB) metropolitan/micropolitan assignment of U.S. counties as updated through the 2005 revisions (Agency for Healthcare Research and Quality 2008a). Geographic region is defined by the US Census Bureau (Agency for Healthcare Research and Quality 2008b). The presence of comorbid conditions was identified using the Clinical Classification Software (CCS) codes that combine similar ICD-9-CM codes (Agency for Healthcare Research and Quality 2014b).

#### Data analysis

Heat stroke visits were weighted to estimate national incidence rates by demographic characteristics and geographic region. Standard errors and corresponding 95% confidence intervals for these national estimates were computed using methodology that accounts for the complex survey design properties for variance estimation (Chen and Gorrell 2008). This method is recommended by HCUP accounting for the complex survey design (Houchens and Elixhauser 2005). All statistical analyses were performed with SAS Version 9.3 (SAS Institute, Cary, NC). This study was approved by the Columbia University Medical Center institutional review board.

### Results

In 2009 and 2010, there were an estimated total of 8,251 ED visits for heat stroke, exclusive of 101,995 ED visits for unspecified heat exhaustion and 39,142 ED visits for other heat-related illnesses. The estimated annual incidence rate of heat stroke was 1.34 visits per 100,000 population (95% CI = 1.23-1.45) (Table 1). Over one-fifth (21.7%) of the ED visits for heat stroke were made by patients aged 70 years and older. The incidence rates for heat stroke ED visits increased with age and were the highest for those aged 80 years or older (4.45 visits per 100,000; 95% CI = 3.73-5.18).

**Table 1:** **Incidence rates and 95**% **confidence intervals (CIs) of heat stroke emergency department visits by patient characteristics, National Emergency Department Sample, United States, 2009 and 2010**

Patient characteristics	Number of patients with heat stroke (weighted)	Incidence per 100000 population	95% CI
Total population	8251	1.34	1.23-1.45
Age(yr)			
0-19	801	0.48	0.40-0.56
20-29	987	1.15	0.93-1.37
30-39	927	1.15	0.97-1.34
40-49	1281	1.47	1.26-1.67
50-59	1342	1.62	1.35-1.89
60-69	1118	1.97	1.61-2.32
70-79	785	2.38	1.98-2.79
80+	1010	4.45	3.73-5.18
Sex			
Male	6022	1.99	1.81-2.16
Female	2219	0.71	0.63-0.79
Region of hospital			
Northeast	890	0.80	0.65-0.96
Midwest	1555	1.16	0.97-1.35
South	3675	1.61	1.43-1.79
West	2132	1.49	1.19-1.78
Patient location			
Metropolitan	6303	1.19	1.08-1.30
Micropolitan	953	1.55	1.24-1.87
Year of visit^a^			
2009	3294	1.07	0.94-1.21
2010	4957	1.61	1.43-1.78

Note. Year of visit was defined as discharge year. There were 5 missing values for sex.

Males accounted for nearly three quarters (73.2%) of all ED visits for heat stroke. Overall, the annual incidence rate of ED visits for heat stroke for males (1.99 visits per 100,000; 95% CI = 1.81-2.16) was 2.8 times the rate for females (0.71 visits per 100,000 95% CI = 0.63-0.79). Residents in the South had the highest incidence rate of ED visits for heat stroke (1.61 visits per 100,000; 95% CI = 1.43-1.79) while those living in the Northeast had the lowest rate (0.80 visits per 100,000; 95% CI = 0.65-0.96). Incidence rates of ED visits for heat stroke were similar between urban areas (1.19 visits per 100,000; 95% CI = 1.08-1.30) and rural areas (1.55 visits per 100,000; 95% CI = 1.24-1.87).

**Figure 1:**
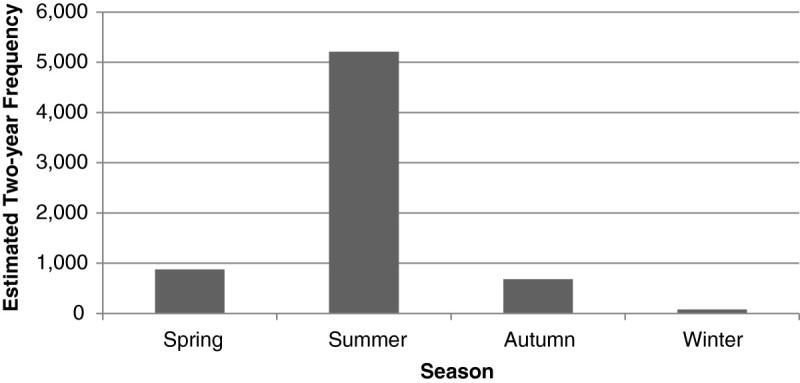
**Estimated two-year frequency of emergency department visits for heat stroke by season, United States, 2009 and 2010.** Note: Monthly and yearly data are not presented as the report of data for any subgroup with 10 or fewer subjects is prohibited by the HCUP NEDS data use agreement. There were 1408 missing values for admission month, 93.7% of which are from hospitals in the southern states.

As expected, ED visits for heat stroke showed an apparent seasonality, with almost two thirds (63.1%) of the visits occurring in the summer from June to August (Figure 1). Exclusive of the heat stroke diagnosis, a total of 53,270 comorbidities were recorded for all heat stroke ED visits. Fluid and electrolyte disorders (CCS code 56) were the most frequent presenting comorbidity (11.4%), followed by acute and unspecified renal failure (CCS code 157, 4.4%), other connective tissue disease (CCS code 211, 4.2%), and essential hypertension (CCS code 98, 4.1%).

Over half (54.6%) of the patients who had an ED visit for heat stroke were admitted to hospitals, 37.8% were treated and released and 3.5% died (including 48 patients who died in the ED and 238 patients who died in hospital). The admission rate and case fatality rate for heat stroke ED visits were much higher than for other ED visits (15.2% and 0.6%, respectively).

### Discussion

This analysis provides valuable information for understanding the epidemiologic patterns of ED visits for heat stroke in the United States. The results indicate that, each year in the United States, there are an estimated 4,126 ED visits for heat stroke, with an annual national incidence rate of 1.34 visits per 100,000 population and a case fatality of 3.5%. In previous studies, the case fatality of classical heat stroke ranged from less than 10% to 64% (Yaqub and Al Deeb 1998; Jimenez-Mejias et al. 1990). The lower case fatality reported in the present study is likely due to the fact that it included only patients treated in the EDs and the most severe cases of heat stroke often result in death at the scene.

This study confirms that the elderly are at heightened risk of heat stroke (Jardine 2007; Semenza et al. 1996). We also found that males are 2.80 times as likely as females to be treated for heat stroke. One previous study reported that being male was a major risk factor of dying from classical heat illness (Stafoggia et al. 2006). In contrast, a study conducted in soldiers found that heat illness risk was significantly greater in women than in men (Carter et al. 2005). Data from a heat wave surveillance system in Korea also showed that women were at a higher risk of dying from heat stroke than men (Na et al. 2013). These seemingly conflicting findings are likely due to the differences in study populations and outcome measures.

Our results indicate that over one-half (63.1%) of all ED visits for heat stroke occur in the summer months of June, July and August whereas 0.9% of them arise in the winter months of December, January and February. This finding is consistent with previous reports that risk for heat illness is a function of ambient temperature, relative humidity, wind speed, and solar radiant heat (Yu et al. 2012; Murakami et al. 2012). In addition, the geographical differences in the incidence rates of ED visits for heat stroke also support the relation between ambient temperature and heat stroke.

Comorbid conditions of heat stroke ED visits reported in our study are similar to those found in previous studies (Jardine 2007; NYC Department of Health and Mental Hygiene 2006; Wheeler et al. 2013). Specifically, the most commonly recorded comorbid condition is fluid and electrolyte disorders, followed by acute and unspecified renal failure, other connective tissue disease, and essential hypertension. It is plausible that some of these comorbid conditions, e.g., fluid and electrolyte disorders and unspecified renal failure, might be in fact the consequences of heat stroke rather than preexisting conditions.

Our study also reveals that heat stroke as a medical emergency is significantly more severe than other emergencies as a whole, with a 2.6-fold increase in the admission rate and a 4.8-fold increase in case fatality. Clinical management of heat stroke often requires aggressive interventions such as cold water immersion and chilled intravenous fluids to low core body temperature as rapidly as possible (Bouchama et al. 2007). Multi-organ dysfunction as a common complication may contribute to the excess case fatality of heat stroke (Pease et al. 2009).

#### Limitations

Our study has several notable limitations. First, the incidence rates of heat stroke are likely to be underestimated since our study was limited to patients presenting to the EDs. Many heat stroke patients, especially those severe cases that progress rapidly, may die before reaching any medical facility. On the other hand, some less severe heat stroke cases might be misclassified as other heat-related illnesses, such as heat syncope or heat exhaustion. Excluded from our analysis were 141,137 ED visits for other heat-related illnesses during the two-year study period. Second, the NEDS does not capture some important demographic information, such as race/ethnicity and marital status. Previous studies indicate that those geographically or socially isolated are particularly vulnerable to heat stroke (Semenza et al. 1996). Finally, the NEDS does not contain a unique identification number for each patient. Thus, it is impossible to account for multiple visits made by a single patient.

## Conclusions

Our study provides valuable data for understanding the magnitude and epidemiologic patterns of heat stroke on a national level. With global warming, there might be a rise in the frequency and severity of extreme heat waves in the United States and elsewhere. Thereby, heat stroke and other heat-related illnesses could become a health threat to a larger population and a medical emergency of increasing importance. It is necessary to strengthen the surveillance of heat-related disorders and develop effective intervention programs to reduce the adverse health consequences of global warming and extreme heat.

## Authors’ original submitted files for images

Below are the links to the authors’ original submitted files for images. Authors’ original file for figure 1

## Abbreviations

ED: Emergency department
ICD-9-CM: International Classification of Diseases, Ninth Revision, Clinical Modification
CI: Confidence interval
NEDS: Nationwide Emergency Department Sample
OMB: Office of Management and Budget
CCS: Clinical Classification Software.
