# Supplementary figures and images for: Emergency Department Visits for Heat Stroke in the United States, 2009 and 2010
Source: Inj Epidemiol. 2014 Apr 24;1(1):8. doi: 10.1186/2197-1714-1-8 (PMC5005673; doi:10.1186/2197-1714-1-8)

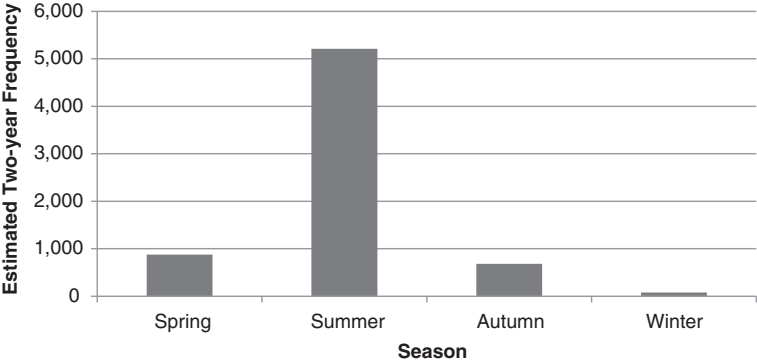

Supplement: Supplementary file 1 — Authors’ original file for figure 1 [file 40621_2013_8_MOESM1_ESM.pdf]
